# Immunogenicity and Protectivity of Sputnik V Vaccine in hACE2-Transgenic Mice against Homologous and Heterologous SARS-CoV-2 Lineages Including Far-Distanced Omicron BA.5

**DOI:** 10.3390/vaccines12101152

**Published:** 2024-10-08

**Authors:** Inna V. Dolzhikova, Amir I. Tukhvatulin, Daria M. Grousova, Ilya D. Zorkov, Marina E. Komyakova, Anna A. Ilyukhina, Anna V. Kovyrshina, Artem Y. Shelkov, Andrey G. Botikov, Ekaterina G. Samokhvalova, Dmitrii A. Reshetnikov, Andrey E. Siniavin, Daria M. Savina, Dmitrii V. Shcheblyakov, Fatima M. Izhaeva, Alina S. Dzharullaeva, Alina S. Erokhova, Olga Popova, Tatiana A. Ozharovskaya, Denis I. Zrelkin, Polina P. Goldovskaya, Alexander S. Semikhin, Olga V. Zubkova, Andrey A. Nedorubov, Vladimir A. Gushchin, Boris S. Naroditsky, Denis Y. Logunov, Alexander L. Gintsburg

**Affiliations:** 1 Federal State Budget Institution “National Research Centre for Epidemiology and Microbiology Named after Honorary Academician N F Gamaleya”, Ministry of Health, Russian Federation, 123098 Moscow, Russia; 2 Federal State Autonomous Educational Institution of Higher Education “I.M. Sechenov First Moscow State Medical University” (Sechenov University), Ministry of Health, Russian Federation, 119991 Moscow, Russia

**Keywords:** vector vaccine, cross-protection, COVID-19 vaccine, hACE2-transgenic mice, immunogenicity

## Abstract

Background: The SARS-CoV-2 virus continuously acquires mutations, leading to the emergence of new variants. Notably, the effectiveness of global vaccination efforts has significantly declined with the rise and spread of the B.1.1.529 (Omicron) variant. Methods: The study used virological, immunological and histological research methods, as well as methods of working with laboratory animals. In this study, we evaluated the Gam-COVID-Vac (Sputnik V), an adenoviral vaccine developed by the N.F. Gamaleya National Research Center for Epidemiology and Microbiology, and conducted experiments on hemizygous K18-ACE2-transgenic F1 mice. The variants studied included B.1.1.1, B.1.1.7, B.1.351, B.1.1.28/P.1, B.1.617.2, and B.1.1.529 BA.5. Results: Our findings demonstrate that the Sputnik V vaccine elicits a robust humoral and cellular immune response, effectively protecting vaccinated animals from challenges posed by various SARS-CoV-2 variants. However, we observed a notable reduction in vaccine efficacy against the B.1.1.529 (Omicron BA.5) variant. Conclusions: Our results indicate that ongoing monitoring of emerging mutations is crucial to assess vaccine efficacy against new SARS-CoV-2 variants to identify those with pandemic potential. If protective efficacy declines, it will be imperative to develop new vaccines tailored to current variants of the virus.

## 1. Introduction

On 30 January 2020, the World Health Organization announced the coronavirus disease 2019 (COVID-19) outbreak as a public health emergency of international concern [1]. A pandemic was declared on 11 March 2020 [2]. Since the emergence and spread of COVID-19, more than 700 million people worldwide have been infected with severe acute respiratory syndrome coronavirus type 2 (SARS-CoV-2) and more than seven million people have died as a result of COVID-19 [3]. In an attempt to reduce the spread of SARS-CoV-2 infection and severe cases of COVID-19, SARS-CoV-2 vaccines have been developed. Vaccine-induced immunity against SARS-CoV-2 has reduced human infections and curtailed the COVID-19 pandemic.

Prevention of SARS-CoV-2 infection can be achieved by targeting the S glycoprotein—a viral envelope protein required to interact with the ACE2 receptor and enter the cell [4]. Blocking S-ACE2 interaction results in decreased viral internalization and replication [5,6,7]. Most vaccines currently being developed and used use the S protein as their primary antigen [8,9]. Since neutralizing antibodies are formed to the surface glycoprotein S after infection or vaccination, key mutations occur in the S gene leading to amino acid changes that allow the virus to evade the immune response. Currently, a number of mutations in the glycoprotein are known to increase the affinity of its binding to the ACE2 receptor and reduce the neutralizing activity of antibodies [10].

The continuous evolution of the SARS-CoV-2 virus has become a major concern throughout the three years of the pandemic [11]. Expression of proofreading 3′-5′ exoribonuclease leads to a reduced mutation rate and the ability to escape from the immune response in coronaviruses. Despite this, many mutations have been discovered in the virus SARS-CoV-2, including mutations in the glycoprotein S, leading to an increase in the severity of COVID-19 and a decrease in the effectiveness of neutralizing antibodies [12]. Since December 2020, several highly infectious SARS-CoV-2 variants of concern have emerged worldwide, including B.1.1.7 (Alpha), B.1.351 (Beta), P.1 (Gamma), B.1.617.2 (Delta) and B.1.1.529 (Omicron) [3,13]. The emergence of the SARS-CoV-2 B.1.1.529 (Omicron) variant may be considered a turning point in the COVID-19 pandemic [14]. The B.1.1.529 (Omicron) variant was first reported in South Africa and spread rapidly, eventually displacing the B.1.617.2 (Delta) variant and becoming the predominant circulating variant [15]. The B.1.1.529 (Omicron) variant has many descendant sublineages, including BA.1, BA.2, BA.3, BA.4 and BA.5 [16]. Initially, B.1.1.529 (Omicron) BA.1 was displaced by the BA.2 variant, which in turn was displaced by its descendants, such as BA.4 and BA.5 [17]. New sublineages of B.1.1.529 (Omicron) have >50 new amino acid substitutions, many of which are in the receptor binding domain (RBD) [18]. The BA.2 virus variant had deletions of H69/V70, which play an important role in ACE2 receptor binding and viral infectivity, while the BA4/BA5 variants characterized by additional spike mutations 69–70 del, L452R, F486V, and an ancestral amino acid at position Q493 [19,20]. In addition, variants BA.2 and BA.4/BA.5 carry new mutations (T19I, A27S, V213G, T376A, D405N, R408S and others), which resulted in virus transmission advantage and evade neutralizing antibodies [21,22]. Numerous amino acid substitutions in the virus resulted in high transmissibility and signs of immune evasion by vaccine-induced neutralizing antibodies and monoclonal antibodies targeting wild-type S glycoprotein [23,24]. Given the rapid evolution of SARS-CoV-2, it remains difficult to develop a universal vaccine that is highly effective against future variants of SARS-CoV-2. Therefore, it is necessary to monitor the effectiveness of existing vaccines and update the antigenic composition of the vaccines if the effectiveness decreases.

Our study was initiated before the global emergence and spread of the various Omicron variants, when only early sublineages of the Omicron virus variants first appeared, so the investigation was limited to the B.1.1.1, B1.1.7 (Alpha), B.1.351 (Beta), B.1.1.28/P.1 (Gamma), B.1.617.2 (Delta) and B.1.1.529 (Omicron) sublineages BA.1, BA.2 and BA.5 virus variants. Gamaleya Research Center (Moscow, Russia) developed a vector-based Sputnik V (Gam-COVID-Vac) vaccine, which is successfully used in 72 countries worldwide. In this study, we used transgenic mice carrying the human ACE2 gene, the most well-characterized and standardized model for studying the effectiveness of vaccines for the prevention of COVID-19. Here we show that the Sputnik V vaccine induces a high-level humoral and cellular immune response, allowing to protect vaccinated animals from challenge with different variants of the SARS-CoV-2 virus. However, we detect a decrease in the vaccine efficacy against variant B.1.1.529 (Omicron).

The data obtained in this study will improve the understanding of the impact of immunization on the cross-efficacy of vaccines against new variants of the virus and thus help guide further modification of the antigenic composition of the vaccine and recommendations for vaccination of humans.

## 2. Materials and Methods

### 2.1. Ethics

The animal experiments were evaluated and approved by the ethics committee of the Gamaleya Center (protocol #9, 16 April 2021). All procedures with SARS-CoV-2 were carried out in approved biosafety level 3 facilities.

### 2.2. Laboratory Animals

All animal experiments were performed in strict accordance with the recommendations of the National Standard of the Russian Federation [25].

The study of immunogenicity used 6–7 weeks old C57BL/6 Gamrc (Gamaleya Research Center, health status SPF): 5 animals for IgG and cellular response, and 10 animals for neutralizing antibody response. The study of protective efficacy used 6–7 weeks old hemizygous K18-ACE2-transgenic F1 mice obtained from crossing transgenic males B6.Cg-Tg(K18-ACE2)2Prlmn/J (Jackson Laboratory, https://www.jax.org/strain/034860, (accessed on 3 October 2024) health status SOPF) and non-transgenic females C57BL/6 Gamrc (Gamaleya Research Center, health status SPF). The mice had free access to water and food. The mice were housed in an ISOcage N system (Tecniplast, Buguggiate, Italy).

### 2.3. Vaccine

The vaccine Gam-COVID-Vac (Sputnik V) consists of a recombinant replication-defective adenovirus type-26 expressing SARS-CoV-2 glycoprotein S (rAd26-S), and a recombinant replication-defective adenovirus type-5 expressing the same glycoprotein (rAd5-S). Both components were developed, manufactured, and stored by N.F. Gamaleya National Research Center of Epidemiology and Microbiology (Moscow, Russia) according to good manufacturing practices. The vaccine dose for mice was 10^9^ viral particles (vp) per dose for both rAd.

### 2.4. Immunization and Further Sampling Procedures

**Figure 1:**
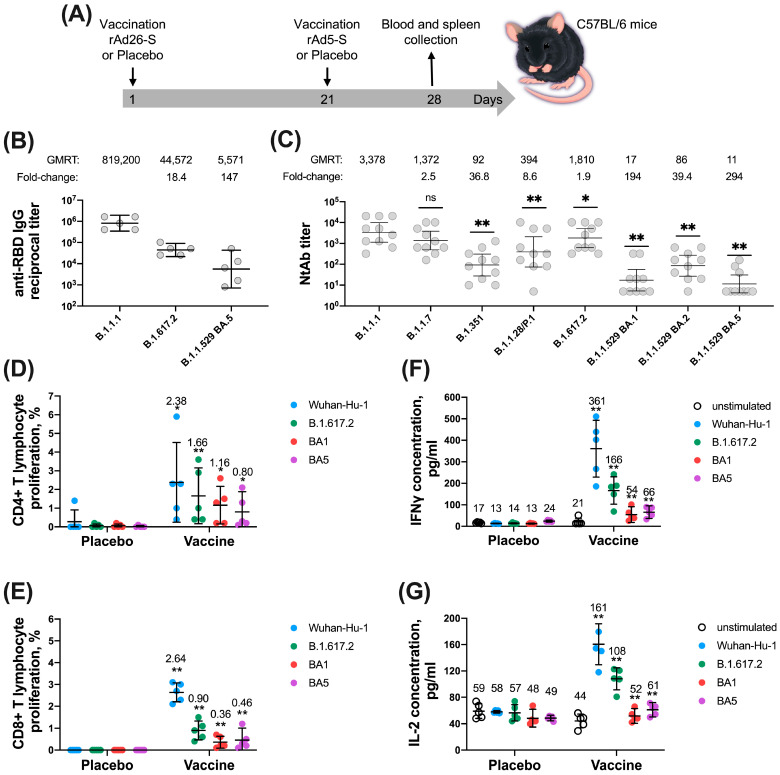
(**A**) Study design. (**B**) RBD-specific IgG antibody levels in the blood sera of Sputnik V-vaccinated mice (*n* = 5) to SARS-CoV-2 variants B.1.1.1, B.1.617.2 (Delta) and B.1.1.529 (Omicron) sublineages BA.1, BA.5. (**C**) Levels of NtAb in the blood sera of Sputnik V-vaccinated mice (*n* = 10) to the SARS-CoV-2 variants B.1.1.1, B1.1.7 (Alpha), B.1.351 (Beta), B.1.1.28/P.1 (Gamma), B.1.617.2 (Delta) and B.1.1.529 (Omicron) sublineages BA.1, BA.2, BA.5. (**D**) The number of proliferating CD4+ T cells derived from spleens of Sputnik V- and PBS-vaccinated mice (*n* = 5 per group) in response to restimulation by recombinant glycoproteins of the B.1.1.1, B.1.617.2 (Delta) and B.1.1.529 (Omicron) sublineages BA.1, BA.5. (**E**) The number of proliferating CD8+ T cells derived from spleens of Sputnik V- and PBS-vaccinated mice (*n* = 5 per group) in response to restimulation by recombinant glycoproteins of the B.1.1.1, B.1.617.2 (Delta) and B.1.1.529 (Omicron) sublineages BA.1, BA.5. (**F**) Concentration of IFN-γ in the medium from splenocytes restimulated with glycoproteins of the B.1.1.1, B.1.617.2 (Delta) and B.1.1.529 (Omicron) sublineages BA.1, BA.5. (**G**) Concentration of IL-2 in the medium from splenocytes restimulated with glycoproteins of the B.1.1.1, B.1.617.2 (Delta) and B.1.1.529 (Omicron) sublineages BA.1, BA.5. Dots represent individual data points. In B-C horizontal lines represent geometric mean titers (values are indicated above each group), and whiskers are 95% CIs. In D-G horizontal lines represent mean (values are indicated above each group), and whiskers are SD. Groups were compared by non-parametric ANOVA (Friedman’s test) with Dunn’s multiple comparison post-test (ns—not significant, * *p* < 0.05, ** *p* < 0.01).

**Figure 2:**
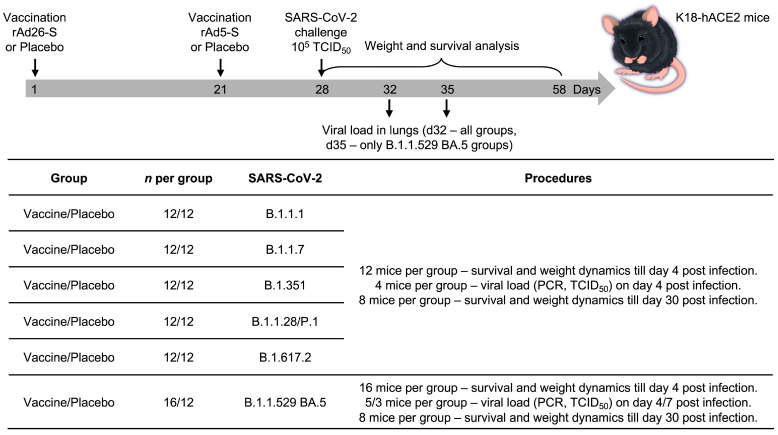
Experiment scheme. K18-hACE2-transgenic mice were immunized twice with vaccine or placebo (PBS buffer). At day 7 after second vaccine/placebo dose mice were challenged with SARS-CoV-2 virus.

In the immunogenicity study mice were divided into 2 groups: vaccine administration and placebo administration (*n* = 5 per group for RBD-specific IgG antibody levels study and for cellular immune response study, *n* = 10 per group for NtAb levels study) (Figure 1). In the protectivity study mice were divided into 12 groups: 6 groups for vaccine administration and 6 groups for placebo administration (*n* = 12 per group for B.1.1.7 (Alpha), B.1.351 (Beta), B.1.1.28/P.1 (Gamma), B.1.617.2 (Delta), *n* = 16 per group for B.1.1.529 (Omicron) sublineage BA.5) (Figure 2). Immunization of animals was carried out intramuscularly according to the scheme recommended for clinical use, twice in the prime-boost regimen: component 1 (rAd26-S) and component 2 (rAd5-S) with 21 days interval. Placebo groups were administered twice with sterile phosphate-buffered saline (PBS). On day 28 after the administration of vaccine/placebo, blood and spleens were taken for further study of the immune response.

### 2.5. ELISA

ELISA protocol was used for the evaluation of anti-RBD antibodies. Serum samples were purified by centrifugation (800× *g*, 10 min). Recombinant SARS-CoV-2 RBD domain of B.1.1.1, B.1.617.2 (Delta) and B.1.1.529 (Omicron) sublineages BA.1, BA.5 (SinoBiological, Beijing, China) was used for overnight coating of 96-well plates (100 ng/well). Plates were washed 5× with washing solution (PBS + 0.1% Tween20, TPBS) and then blocked with blocking solution (TPBS with 5% non-fat dry milk). Serum samples were serially diluted in blocking solution and added in wells, then plates were incubated at 37 °C for 1 h. After washing the plates anti-mouse total IgG secondary HRP-conjugated antibodies (1:5000 dilution) (Abcam, Cambridge, UK) in blocking solution were added and plates were incubated at 37 °C for 1 h. After washing the substrate TMB was added and plates were incubated at 20–25 °C, the reaction was stopped with 4 M H_2_SO_4_. A colorimetric signal was measured at 450 nm using a Multiscan FC spectrophotometric plate reader (Thermo Fisher Scientific, Waltham, MA, USA) 30 min after the addition of stop solution.

### 2.6. SARS-CoV-2 Preparation

The following SARS-CoV-2 variants were used in the assay: B.1.1.1 (hCoV-19/Russia/Moscow_PMVL-1/2020), B.1.1.7 (hCoV-19/Netherlands/NoordHolland_20432/2020), B.1.351 (hCoV-19/Russia/SPE-RII-27029S/2021), B.1.1.28/P.1 (hCoV-19/Netherlands/NoordHolland_10915/2021), B.1.617.2 (hCoV-19/Russia/SPE-RII-32758S/2021) and B.1.1.529 BA.5 (hCoV-19/Russia/SPE-RII-25357S/2022). SARS-CoV-2 variants were propagated in Vero E6 cells in DMEM with 2% HI-FBS, harvested after 72 h, aliquoted, titrated on Vero E6 cells and stored at −80 °C. The virus titer was determined on Vero E6 cells using a 50% tissue culture infectious dose (TCID_50_) assay. Serial 10-fold dilutions of the virus stock were prepared in DMEM with 2% HI-FBS and in the volume of 100 μL were added to Vero E6 cells in a 96-well plate in 8 repeats. The cells were incubated at 37 °C in 5% CO_2_ for 96–120 h and scored visually for cytopathic effect. The TCID_50_ titer was calculated by the Spearman–Kerber method.

### 2.7. Neutralization Assay

NtAb titer was determined by microneutralization assay in a 96-well plate. Serum samples were inactivated (56 °C, 30 min), serially diluted in complete DMEM supplemented with 2% HI-FBS, mixed at a 1:1 ratio with 100 TCID_50_ (50% tissue culture infectious dose) of each SARS-CoV-2 variant in total volume of 100 μL, incubated at 37 °C for 1 h, added to Vero E6 cell monolayer and incubated for 96 h. The cytopathic effect (CPE) of the virus on the cell was assessed visually. Neutralization titer was defined as the highest serum dilution without any CPE in two of three replicable wells. Samples with no neutralization at starting dilution points were attributed to two-fold lower values.

### 2.8. Study of Cellular Immune Response

Splenocytes were purified by density gradient centrifugation (400× *g*, 30 min) using Ficoll 1.09 g/mL (PanEco, Moscow, Russia), washed in PBS, counted, stained using CellTrace™ carboxyfluorescein diacetate succinimidyl ester (CFSE) Cell Proliferation Kit (Invitrogen, Carlsbad, California, USA) according to the manufacturer’s instructions, seeded in duplicates in 96-well plates at 2 × 10^5^ cells per well and stimulated with 5 µg/mL recombinant SARS-CoV-2 S glycoprotein of B.1.1.1, B.1.617.2 (Delta) and B.1.1.529 (Omicron) sublineages BA.1 and BA.5 (SinoBiological, Beijing, China). Cells and cell-free media samples were collected 96 h after treatment. Cells were stained with DAPI (1 µg/mL) to exclude dead cells and anti-CD3 (PE-Cy7, clone 145-2C11), anti-CD4 (PE, clone RM4-5), and anti-CD8 (APC, clone 53-6.7) antibodies for 40 min at 4 °C in Staining Buffer (BD Biosciences, Franklin Lakes, NJ, USA). Proliferating CD4+ or CD8+ T-lymphocytes were expressed as a percentage of cells in the final culture that divided at least once (referring to “Fraction diluted” statistic) [26]. To evaluate the prominent cell-mediated Th1 response, the IL-2 and IFN-γ cytokine levels were analyzed in cell-free media samples using a mouse 23-plex bead-based Bio-Plex Pro Kit (Bio-Rad Laboratories, Hercules, CA, USA) according to the manufacturer’s instructions.

### 2.9. Animal Challenge

At day 7 after boost vaccination (day 28 after prime vaccination), animals were challenged with different SARS-CoV-2 variants (B.1.1.1, B.1.1.7, B.1.351, B.1.1.28/P.1, B.1.617.2 and B.1.1.529 BA.5) intranasally at a dose of 10^5^ TCID_50_. Four animals per group were euthanized on day 4 post-challenge for macroscopic analysis of lung damages and for determination of viral load in lungs. All other animals in groups were monitored for weight loss and survival. Animals with weight loss of more than 20% from initial weight were euthanized before the end of the study.

### 2.10. Determination of Viral Load in Lungs

A total of 10% lung homogenates in DMEM with 2% heat-inactivated FBS were prepared on day 4 after challenge in MPbio FastPrep-24 (MP Biomedicals, Irvine, CA, USA), the homogenates were centrifuged at 12,000× *g* for 10 min and supernatant were used for further analysis. The infectious virus titer was determined as described above on Vero E6 cells in a 96-well plate in 4 repeats after 120 h incubation.

### 2.11. Histopathological Analysis of the Lungs

Lung samples were fixed in 10% buffered neutral formalin (Soluform™, JLS-Chemical, Saint Petersburg, Russia) at +4 °C, dehydrated in isoprep. Impregnated in HISTOMIX^®^ (all BioVitrum, Saint Petersburg, Russia) using Microm STP 120 (Thermo Scientific, Waltham, MA, USA) and embedded in HISTOMIX^®^ (BioVitrum, Saint Petersburg, Russia) using HistoStar (Thermo Scientific, Waltham, MA, USA). Histological sections–4–5 µm thick were prepared on a Finesse ME+ microtome (Thermo Scientific, Waltham, MA, USA), stained with hematoxylin and eosin, and mounted under a coverslip in Vitrogel mounting medium (all BioVitrum, Saint Petersburg, Russia). Epson Perfection V600 (9600 dpi) was used to obtain scans of lung tissue. Frontal sections of the lungs were analyzed on an Imager.Z1 (ZEISS, Oberkochen, Germany), and images were obtained at 20x objective magnification using an AxioCam MRc 5 (ZEISS, Oberkochen, Germany).

The degree of pathological changes in lung tissue was assessed according to previously used methods [27]. Briefly, the analysis was carried out using three parameters: (1) acute lung injury (ALI) score (from 0 to 1)—10 random fields of view for each animal; (2) peribronchiolar inflammation score (from 0 to 5)—10 random bronchioles for each mouse; (3) perivascular inflammation score (from 0 to 5)—10 random vessels for each animal. We assessed each parameter in accordance with the specified criteria (Table 1 and Table 2). To obtain the final ALI score, we used the following formula: score = [(20 × A) + (14 × B) + (7 × C) + (2 × D)]/86.

### 2.12. Statistical Analysis

All statistical calculations were performed in the GraphPad Prism 9. The normality of data distribution was evaluated in the d’Agostino–Pearson test. A comparison of unpaired samples was performed by the Mann–Whitney test. Multiple data were compared by non-parametric ANOVA (Friedman’s test) with Dunn’s multiple comparison post-test. Survival curves were compared using the Log-rank (Mantel–Cox) test.

## 3. Results

### 3.1. Sputnik V Prime-Boost Immunization Results in Broad Cross-Reactive Humoral and Cellular Immune Response across Different VOCs

To evaluate the breadth and cross-reactivity of post-vaccinal immune response to different virus variants, C57BL/6 mice were vaccinated consequently with two vaccine components with a 21-day interval between the prime and boost immunization. On day 28 after prime vaccination, blood was collected to evaluate titers of RBD antigen-binding and virus-neutralizing antibodies (NtAb) as well as splenocytes for evaluation of cell-mediated immune response: percentages of S-stimulated proliferating CD4+, CD8+ T cells and concentration of IFN-γ, IL-2 cytokines (Figure 1A).

High levels of anti-Receptor Binding Domain (RBD) IgG antibodies as well as virus neutralizing antibodies were detected against the homologous B.1.1.1 (Wuhan SARS-CoV-2 virus) (GMRTs 819200 and 3378, respectively), while we also detected a cross-reactivity of studied parameters of the humoral immune response against other variants of the SARS-CoV-2 virus: B1.1.7 (Alpha), B.1.351 (Beta), B.1.1.28/P.1 (Gamma), B.1.617.2 (Delta) and B.1.1.529 (Omicron) sublineages BA.1, BA.2, BA.5 (Figure 1B,C). The greatest decrease in the levels of both RBD-specific IgG and NtAbs antibodies, but still discernible compared with the level of unvaccinated mice were detected in the B.1.1.529 (Omicron) sublineage BA.5: 147- and 294-fold decrease for RBD-specific IgG and NtAb, respectively.

Analysis of the cellular response of vaccinated animals revealed the most significantly higher percentages of proliferating CD4+ as well as CD8+ T (CD3+) cells following restimulation with Wuhan S antigen (2.38 and 2.64%, respectively), while stimulation with S proteins derived from heterologous B.1.617.2 (Delta) and B.1.1.529 (Omicron) sublineages BA.1 and BA.5 variants resulted in less portion of CD4+ (1.66, 1.16 and 0.80%, respectively) and CD8+ (0.90, 0.36 and 0.46%, respectively) T cells. The highest values of IFN-γ and IL-2 cytokines in the medium from splenocytes were detected upon stimulation with S protein from Wuhan-Hu-1 SARS-CoV-2 strain: 361 and 161pg/mL, respectively. Stimulation with heterologous glycoproteins of the B.1.617.2 (Delta) and B.1.1.529 (Omicron) sublineages BA.1, BA.5 resulted in a significant decrease in cytokine production (folds). However, the low levels of proliferating CD4+, CD8+ T cells and cytokines were statistically higher than those observed in sham-vaccinated (PBS) mice.

### 3.2. Sputnik V Prime-Boost Immunization Results in Protection of Lung Pathology Caused by Different VOCs

Next, we analyzed the protective efficacy of the Sputnik V vaccine on K18-hACE2-transgenic mice against different SARS-CoV-2 variants. Mice were vaccinated consequently with two vaccine components with a 21-day interval between the prime and boost immunization. On day 28 after prime vaccination mice were infected with 1 × 10^5^ TCID50 of SARS-CoV-2 variants B1.1.7 (Alpha), B.1.351 (Beta), B.1.1.28/P.1 (Gamma), B.1.617.2 (Delta) and B.1.1.529 (Omicron) sublineage BA.5 via the intranasal route (Figure 2). At day 4 after infection, a part of the infected animals (*n* = 4 per group for B1.1.7 (Alpha), B.1.351 (Beta), B.1.1.28/P.1 (Gamma), B.1.617.2 (Delta), *n* = 5 per group for B.1.1.529 (Omicron) sublineage BA.5) were euthanized for assessment of virus-associated lung pathology (Figure 2). Since the Omicron BA.5 turned out to be less pathogenic for transgenic mice in terms of lethality, we used a larger number of animals to analyze the viral load in the lungs (*n* = 5 on day 4) and the lung tissue damage than in other groups (*n* = 5 on day 4, *n* = 3 on day 7) (Figure 2). Another part of the same animal groups (*n* = 8 per group) was monitored to evaluate the survival rate.

**Figure 3:**
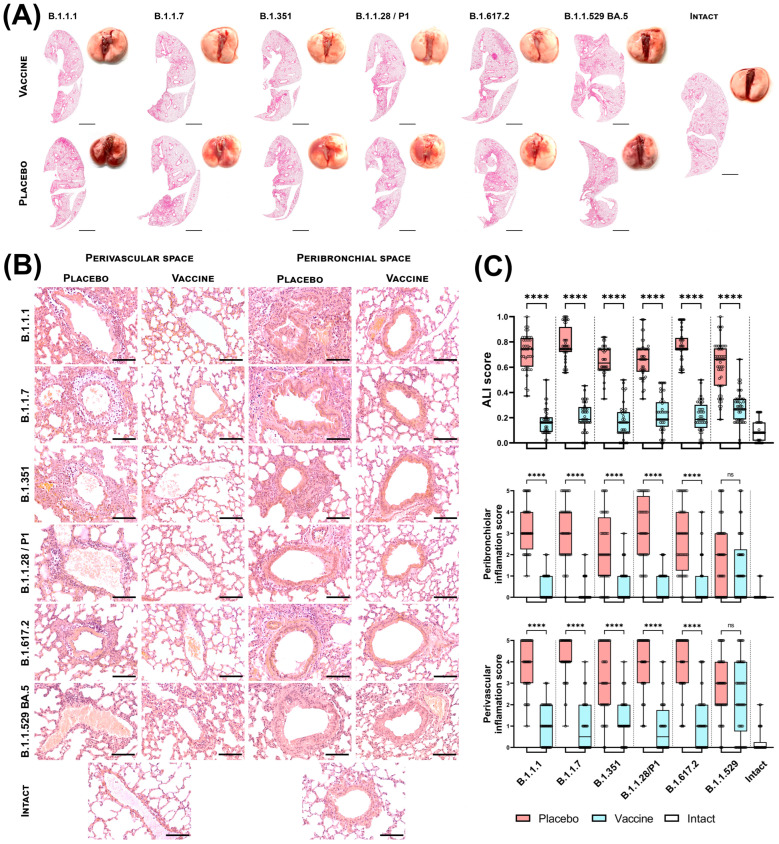
(**A**) Macrophotographs and slide scans of the lungs of unvaccinated (bottom row) and vaccinated with Sputnik V (top row) K18-hACE2 mice challenged with SARS-CoV-2; hematoxylin and eosin (H&E) staining; bar = 2 mm. (**B**) Histopathological analysis of K18-hACE2 mouse lungs: perivascular space (left column) and peribronchiolar space (right column) of mouse lungs; H&E; bar = 100 µm. (**C**) Acute lung injury (ALI) score; 5-point score for peribronchiolar inflammation; 5-point assessment of perivascular inflammation. The boxes show the interquartile range, the whiskers show the range from minimum to maximum, and the horizontal line shows the median value. Each point on the graph corresponds to 1 field of view/1 vessel/1 bronchiole, 10 points were measured for each mouse, the data are summarized for all animals of one experimental group. The red color of the boxes corresponds to the placebo group, the light blue color represents the vaccinated group, and the white color of the boxes to the group of intact mice. Significant differences between vaccinated Sputnik V and non-vaccinated mice were measured using the two-tailed Mann–Whitney test (**** *p* < 0.0001, ns—not significant).

We found that unvaccinated K18-hACE2 transgenic mice were highly susceptible to all SARS-CoV-2 variants. (Figure 3). Lung macroscopy of infected animals showed multiple damages and hemorrhages in the lungs of mice from placebo groups, while the lungs of vaccinated animals had no visual signs of damage (Figure 3A). Slide scans of mouse lungs show diffuse areas of tissue compaction accompanied by inflammation (Figure 3A).

Histological analysis showed congestion of blood vessels, thickening of interalveolar septa and intravascular leukocytosis in the lungs of unvaccinated mice.

The difference between vaccinated and placebo mice is most clearly noticeable in the state of the bronchial tree and the presence of an inflammatory process in the lungs. The lungs of placebo mice demonstrate massive infiltration of the perivascular zone with leukocytes (mainly lymphocytes and neutrophils) (Figure 3B) with foci of infiltration extending to fill several fields of view. The most severe inflammation was found in placebo-treated groups challenged with B1.1.7 (Alpha), B.1.351 (Beta) and B.1.617.2 (Delta) variants of SARS-CoV-2. We observed the lowest degree of damage to the perivascular area among placebo mice upon infection with B.1.1.529 (Omicron) sublineage BA.5.

In vaccinated mice, vasculitis in the lungs is either absent or occurs in small amounts in animals infected with B1.1.7 (Alpha), B.1.351 (Beta) and B.1.617.2 (Delta) variants of SARS-CoV-2. (Figure 3B).

While among vaccinated mice, it was infection with B.1.1.529 (Omicron) sublineage BA.5 that led to the most pronounced perivascular inflammation. The bronchial tree of placebo mice is characterized by desquamation of the bronchial epithelium and filling of the lumen of the bronchi and bronchioles with macrophages and postcellular structures. Vaccinated animals show only a rare increase in the number of bronchial macrophages. In addition, in the lungs of placebo-treated animals, we identified an extensive peribronchiolar infiltrate consisting mainly of lymphocytes. While the interstitium of the bronchi and bronchioles of vaccinated mice shows only single leukocytes.

According to the ALI criterion, SARS-CoV-2 causes severe lung damage in mice. All analyzed variants of SARS-CoV-2 demonstrated a clear pathological effect on the lung tissue, which was expressed mainly in neutrophilic infiltration of the alveolar septa and edema of the interalveolar septa, while vaccination with Sputnik V minimized SARS-CoV-2-induced pathological changes, maintaining statistical significance between all treatment groups (Figure 3C, top graph). We also obtained similar results when analyzing the state of the peribronchiolar and perivascular space. SARS-CoV-2 infection promotes the formation of foci of inflammatory infiltration both around the bronchioles (Figure 3C, middle graph) and, especially, around the lung vessels of mice (Figure 3C, lower graph). In vaccinated animals, isolated foci of inflammation occur, but, in general, there is a tendency to preserve normal tissue architecture, as close as possible to that of intact animals.

**Figure A1:**
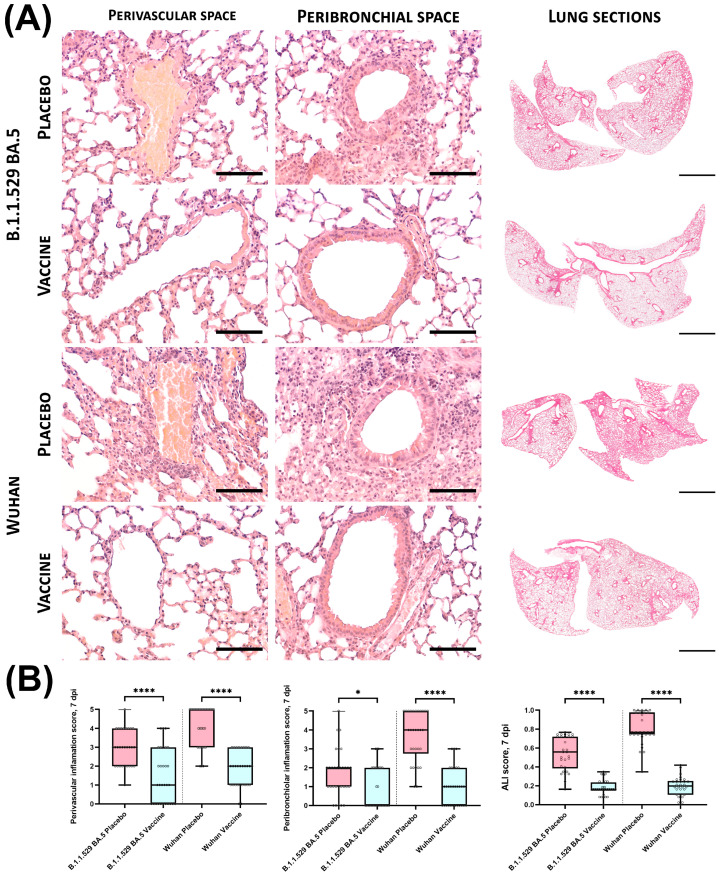
Histopathological analysis of the lungs of K18-hACE2 mice 7 days after infection with SARS-CoV-2 (B.1.1.529 (Omicron) sublineage BA.5 or B.1.1.1 (Wuhan)) with Sputnik V vaccination (vaccine group) or without vaccination (placebo group). (**A**) Architecture of the perivascular space (left column) and peribronchiolar space (middle column), H&E, bar = 100 µm; architecture of a full mouse lung section (right column), H&E, bar = 2 mm. (**B**) 5-point assessment of perivascular inflammation; 5-point score for peribronchiolar inflammation; acute lung injury (ALI) score. The boxes show the interquartile range, the whiskers show the range from minimum to maximum, and the horizontal line shows the median value. Each point on the graph corresponds to 1 field of view/1 vessel/1 bronchiole, 10 points were measured for each mouse, the data are summarized for all animals of one experimental group. The red color of the boxes corresponds to the placebo group, the light blue color represents the vaccinated group. Significant differences between vaccinated Sputnik V and non-vaccinated mice were measured using the two-tailed Mann-Whitney test (**** *p* < 0.0001, * *p* < 0.0332).

Additionally, a comparative analysis of mouse lung tissue was carried out 7 days after infection with either B.1.1.1 or B.1.1.529 (Omicron) sublineage BA.5 (Figure A1A). According to histological analysis, B.1.1.1 infection leads to multifocal pulmonary edema, alveolar and stromal inflammation, vascular congestion and the formation of a massive perivascular and peribronchiolar inflammatory infiltrate. Infection with B.1.1.529 (Omicron) sublineage BA.5 has a less pathological effect on the lung tissue of mice: almost the same vascular congestion, the interalveolar septa are diffusely thickened, but areas of inflammation are less common inside the lung, mainly perivascular mononuclear infiltration is pronounced, peribronchiolar infiltration is less common. The lungs of all vaccinated mice (with both types of infection) were predominantly clear; among the pathological manifestations, rare thickening of the interalveolar septa, rare vascular congestion and the presence of single leukocytes in the perivascular space can be noted. The results of morphometry, especially ALI analysis, confirmed the results of visual analysis (Figure A1B)—the degree of damage to the lung tissue of placebo mice was significantly higher than that of vaccinated mice; however, mice infected with B.1.1.1 showed more severe lung damage than mice infected B.1.1.529 (Omicron) sublineage BA.5.

### 3.3. Sputnik V Prime-Boost Immunization Results in 100% Survival after Lethal SARS-CoV-2 Challenge with Different VOCs

**Figure 4:**
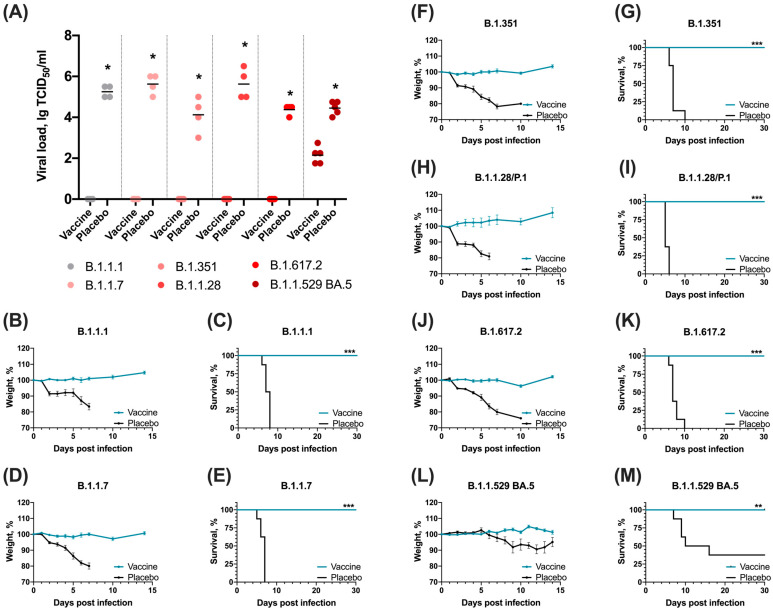
(**A**) Viral load (lgTCID_50_/_mL_) in lungs of vaccinated with Sputnik V (Vaccine) and non-vaccinated (placebo) mice at day 4 after SARS-CoV-2 challenge (*n* = 4 per Wuhan-Delta groups, *n* = 5 per Omicron BA.5 group). Significant differences between vaccinated Sputnik V and non-vaccinated mice were measured using the two-tailed Mann-Whitney test (* *p* < 0.05). (**B**,**D**,**F**,**H**,**J**,**L**) Weight dynamics (mean ± SEM) in Vaccine and Control groups after challenge with SARS-CoV-2 variants B.1.1.1 (**B**), B.1.1.7 (**D**), B.1.351 (**F**), B.1.1.28/P.1 (**H**), B.1.617.2 (**J**), B.1.1.529 BA.5 (**L**) (*n* = 8/per group). Survival (%) in Vaccine and Control groups after challenge with SARS-CoV-2 variants B.1.1.1 (**C**), B.1.1.7 (**E**), B.1.351 (**G**), B.1.1.28/P.1 (**I**), B.1.617.2 (**K**), B.1.1.529 BA.5 (**L**,**M**) (*n* = 8/per group). Significant differences between vaccinated Sputnik V and non-vaccinated mice were measured using the Log-rank (Mantel–Cox) test (*** *p* <0.001, ** *p* < 0.01, * *p* < 0.05).

On day 28 after prime vaccination mice were infected with 10^5^ TCID_50_ of different variants of SARS-CoV-2 virus. Infectious viral titers at day 4 post-infection (dpi) were measured in the lungs (placebo and vaccinated groups) of infected animals. Comparing titers of infectious virus in the lungs, animals vaccinated with Sputnik V showed the highest level of protection. In vaccinated animals, there was a complete absence of virus reproduction in the lungs, compared to the placebo group. Importantly, a challenge with B.1.1.529 (Omicron) sublineage BA.5 led to virus replication in lungs (on day 4), but viral titer in vaccinated animals was reduced by 3 Log_10_ compared to the placebo group (*p* < 0.001, Figure 4A).

Despite the detected viral titer in the lungs of vaccinated animals infected with B.1.1.529 (Omicron) sublineage BA.5, we did not observe clinical signs of infection, weight loss or death in this group. Analysis of weight showed no significant weight loss in the vaccine group after the challenge with all studied SARS-CoV-2 variants, while in placebo groups weight loss was detected at day 2 after the challenge and progressed in time (Figure 4). Survival rates showed complete protection by Sputnik V vaccine in K18-hACE2-transgenic mice against lethal challenge with SARS-CoV-2 variants B1.1.7 (Alpha), B.1.351 (Beta), B.1.1.28/P.1 (Gamma), B.1.617.2 (Delta) and B.1.1.529 (Omicron) sublineage BA.5 (Figure 4).

## 4. Discussion

The emergence and spread of new variants of the SARS-CoV-2 virus can lead to an increase in the incidence of COVID-19 around the world. In this connection, it is necessary to analyze the effectiveness of the vaccines used. During the three years of the COVID-19 pandemic, the Gamaleya Center has operated a laboratory monitoring system for the effectiveness of the Gam-COVID-Vac vaccine (Sputnik V). Monitoring is being conducted in two directions: analysis of the NtAb levels in the blood serum of vaccinated people [28,29], and analysis of the vaccine effectiveness against new SARS-CoV-2 variants in animal models of infection.

Here we present the results of an analysis of the effectiveness of the Sputnik V vaccine against different variants of the SARS-CoV-2 virus (B1.1.7 (Alpha), B.1.351 (Beta), B.1.1.28/P.1 (Gamma), B.1.617.2 (Delta) and B.1.1.529 (Omicron) sublineage BA.5) in animals.

Analysis of post-vaccination immunity demonstrates a significant decrease in the level of specific antibodies to the VOC variants, starting with the B.1.351 (Beta) variant, which correlates with the data obtained by analyzing the level of neutralizing antibodies in the blood sera of vaccinated volunteers [28,29]. It is important to note that despite a significant reduction in NtAb to the B.1.351 (Beta) and B.1.1.28/P.1 (Gamma) variants, we detect complete protection of vaccinated animals (analysis of lesions in the lungs, viral load in the lungs, survival). We detected the most critical decrease in NtAb for the B.1.1.529 (Omicron) BA.5 variant, and infection of animals led to the development of a breakthrough infection: in vaccinated animals, virus reproduction was detected in the lungs, but the viral load was significantly lower than in the control group of animals (about 2 lg TCID_50_). These results are consistent with previously published data showing significant immune escape of BA.5 subvariant against antibodies from vaccinated individuals or individuals infected with BA.1 or BA.2 [17,22,30]. Despite the development of infection, the animals showed no signs of infection and no deaths were recorded after infection with the B.1.1.529 (Omicron) BA.5 variant. Considering the critical decrease in the level of NtAb to the B.1.1.529 (Omicron) BA.5 variant and the protection of animals from lethal infection, it is likely that the cellular component of immunity plays a significant role in the formation of a protective cross-protective immune response [31].

At the same time, we found high antigen-specific reactions of the cellular part of immunity. Adenoviral vector vaccines are shown to elicit a prominent cell-mediated Th1 response which plays an important protective role against viral pathogens [32,33]. Describing the cytokine repertoire of T cells followed by vaccination IFN-γ and IL-2 serve as well-studied markers of functioning Th1 polarized cells [34]. The humoral part of the adaptive immune response was evaluated by measuring titers of antigen-specific IgGs and NtAbs against live viruses. We deliberately used well-defined almost ultimate criteria for both humoral and cellular postvaccinal immune response to concisely show changes in magnitude of immune response across SARS-CoV-2 variants after Sputnik V immunization. We also overlayed survival rates in ACE2 mice across variants of SARS-CoV-2 to indicate how the decrease in humoral and cellular immune responses corresponds with vaccine protection.

The observed decrease in the efficacy of vaccines against the Omicron BA.5 variant may be due to several factors. First of all, the appearance of a combination of the amino acid substitutions L452R, F486V and R493Q in the receptor-binding domain, leads to the evasion of neutralizing antibodies and to an increase in binding affinity to the ACE2 receptor [35]. Overall, three main strategies for immune evasion are known for the Omicron variant: disruption of the humoral immune response; interruption of the cellular immune response; and disruption of innate immune responses, such as induction of cytokine storms and increase in apoptosis-associated proteins [36]. The BA.5 sublineage has shown a significant increase in transmissibility, leading to increased morbidity (including morbidity among vaccinated individuals) and displacement of all previous variants by July 2022 [3,37].

According to the epidemiological efficacy of all used vaccines worldwide, obtained as a result of a meta-analysis, the efficacy in protecting against infection was more than 86% for the Alpha variant, more than 70% for the Beta, Gamma and Delta variants and 23% for the first sublineages of the Omicron variant. Despite the decrease in efficacy against infection, protection against severe disease and COVID-associated deaths remained high during the period of spread of all variants [38]. According to the results of our previous studies of virus-neutralizing antibodies in the blood sera of volunteers vaccinated with Sputnik V, no decrease was found in relation to the Alpha variant, but a 3.1 times decrease in the neutralizing activity of antibodies to the Beta variant was detected, 2.8 times to the Gamma variant, 2.5 times to the Delta variant [28] and more than 8 times to the Omicron sublineage BA.1 [29]. At the same time, studies of the protective efficacy of the vaccine on the COVID-19 model in animals, described in the current manuscript, showed 100% protection of vaccinated animals from infection caused by the above variants. Apparently, the results of the neutralization assay are comparable with the results of the efficacy in protecting against infection, and the results of the analysis of efficacy in animals are comparable with the results of the efficacy in protecting against severe disease and death.

## 5. Conclusions

The decrease in vaccine effectiveness, detected in laboratory monitoring, is confirmed by epidemiological analysis data—with the spread of the Omicron variant, the incidence of COVID-19 has increased significantly both among the unvaccinated population and among the vaccinated. The emergence of new variants of the virus poses the task of constant monitoring of the effectiveness and updating of the antigenic composition of COVID-19 vaccines. Thus, in the first half of 2023, the FDA and WHO published recommendations on the need to switch to the antigenic composition to XBB sub-lineages in COVID-19 vaccines in order to form protective immunity in the population. At the Gamaleya Center, the Sputnik V vaccine was updated for the XBB variant. The updated vaccine finished successfully clinical trials and was introduced into civilian circulation to protect the population from actual variants of the SARS-CoV-2 virus.

## Figures and Tables

**Table 1:** Criteria for ALI scoring system.

Parameter		Score		
		0	1	2
A	Neutrophils in the alveolar space	-	1–5	>5
B	Neutrophils in the interstitial space	-	1–5	>5
C	Proteinaceous debris filling the airspaces	-	1	>1
D	Alveolar septal thickening	<2×	2×–4×	>4×

**Table 2:** Criteria for assessing peribronchiolar and perivascular inflammation.

Score	The Degree of Manifestation of Inflammation
0	Absolute absence of inflammatory infiltrate
1	The presence of single leukocytes
2	The presence of 1–2 groups of leukocytes
3	Infiltration of half of the bronchiole/vessel perimeter
4	Infiltration of most of the bronchiole/vessel perimeter
5	Infiltration of the entire perimeter of the bronchiole/vessel with radial strands of leukocytes

## Data Availability

The data presented in this study are available on request from the corresponding author.
